# Functional biomaterials for osteoarthritis treatment: From research to application

**DOI:** 10.1002/SMMD.20220014

**Published:** 2022-12-27

**Authors:** Yang Lei, Qingfei Zhang, Gaizheng Kuang, Xiaochen Wang, Qihui Fan, Fangfu Ye

**Affiliations:** ^1^ Oujiang Laboratory (Zhejiang Lab for Regenerative Medicine, Vision and Brain Health) Wenzhou Institute University of Chinese Academy of Sciences Wenzhou Zhejiang China; ^2^ Beijing National Laboratory for Condensed Matter Physics Institute of Physics Chinese Academy of Sciences Beijing China; ^3^ School of Physical Sciences University of Chinese Academy of Sciences Beijing China

**Keywords:** biomaterials, exosome therapy, hydrogel, osteoarthritis, stem cell therapy

## Abstract

Osteoarthritis (OA) is a common disease that endangers millions of middle‐aged and elderly people worldwide. Researchers from different fields have made great efforts and achieved remarkable progress in the pathogenesis and treatment of OA. However, there is still no cure for OA. In this review, we discuss the pathogenesis of OA and summarize the current clinical therapies. Moreover, we introduce various natural and synthetic biomaterials for drug release, cartilage transplantation, and joint lubricant during the OA treatment. We also present our perspectives and insights on OA treatment in the future. We hope that this review will foster communication and collaboration among biological, clinical, and biomaterial researchers, paving the way for OA therapeutic breakthroughs.

## Graphical abstract

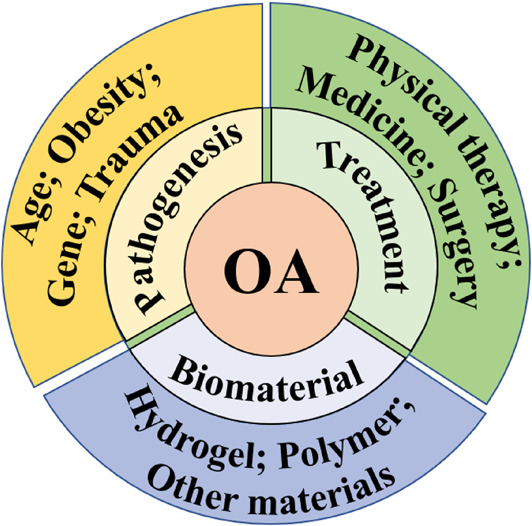

1

**Key points**

- We discuss the pathogenesis of osteoarthritis and summarize the current clinical therapies.
- We introduce different biomaterials and biotechnologies for osteoarthritis treatment.
- This review will foster communication and collaboration among biological, clinical, and biomaterial researchers.

## 2 INTRODUCTION

**FIGURE 1:**
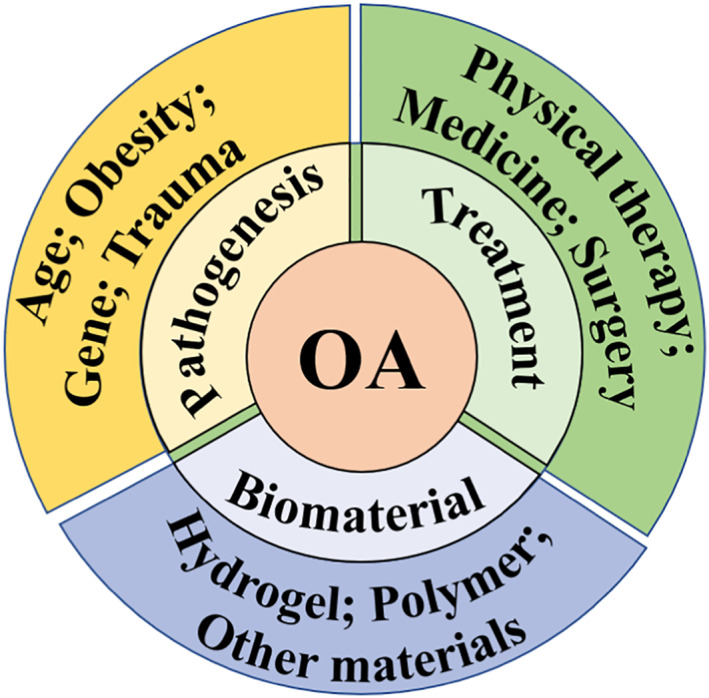
The main content of this review includes Osteoarthritis (OA) pathogenesis, clinical treatment strategies, and functional biomaterials applied in OA therapy.

Osteoarthritis (OA) is a common degenerative disease with the characteristics of cartilage degeneration, osteophyte formation, and synovitis, endangering millions of middle‐aged and older people worldwide.^1^, ^2^, ^3^, ^4^ The increasing prevalence of obese patients also exacerbates the incidence of OA in humans.^5^, ^6^ Joint pain, stiffness, and limited mobility are typical clinical symptoms of OA, which reduce the quality of life and increase the financial burden on the patients, their families, and society. Physical, chemical, and surgical treatments are common OA treatment strategies aimed at pain relief, lubrication enhancement, and joint function improvement.^7^, ^8^, ^9^ However, these standard therapies for OA have limited efficacy. Nowadays, much research progress has been made in the understanding of OA pathogenesis, and various novel OA biotherapy strategies have emerged. Furthermore, the emerging functional biomaterial‐based therapies have shown great potential for the treatment of OA. The combination of multidisciplinary and multimodal approaches will deepen the understanding of OA pathogenesis and facilitate the development of targeted drugs and cutting‐edge biomaterial‐based therapies, thus providing promising ways to cure OA in the future. Most of the previous reviews detailed the significant developments from the perspective of a single discipline, such as biology, medicine, and materials science. Here, we review the research progress in OA from a multidisciplinary perspective, which may inspire and promote the collaboration of researchers in different fields (Figure 1).

In this review, we first introduce OA pathogenesis caused by genetic, epigenetic, and environmental factors. Then, we analyze the commonly applied treatment strategies for OA, including nonpharmacologic, pharmacologic, and surgical therapies. Next, the advances of functional biomaterials applied in OA treatments, such as hydrogel and nanomaterial, are discussed. Finally, we present the remaining challenges and conclude with an outlook of functional biomaterials for future OA treatment. We expect that by better understanding OA pathophysiology and the present biomaterials for treating OA, material scientists and physicians will be able to create novel OA treatments in the future.

## 3 PATHOGENESIS OF OA AND CLINICAL TREATMENT

### 3.1 Cause and pathogenesis of OA

**FIGURE 2:**
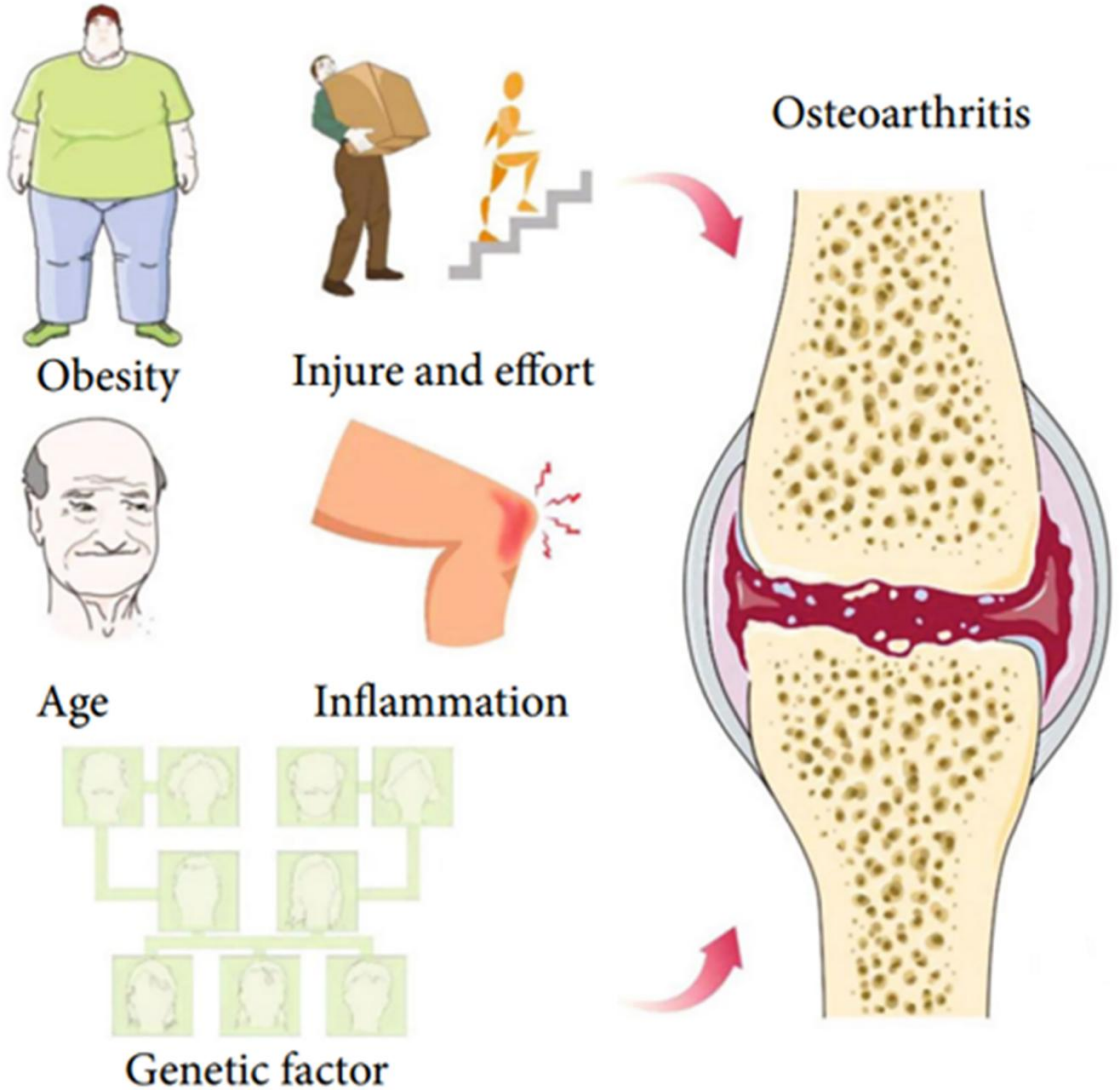
The inducing factors of Osteoarthritis include obesity, age, genetic factor, joint trauma, inflammation, etc. Reproduced under terms of the CC‐BY license.^16^ Copyright 2021, The Authors, published by Hindawi Publishing Corporation.

Different pathophysiological processes are involved in the pathogenesis of OA. According to the etiology, OA can be divided into primary and secondary OA.^10^ Primary OA is closely related to genetic and physical factors, which is more common in middle‐aged and older people.^11^, ^12^ While secondary OA is often caused by joint infection, trauma, congenital or genetic diseases, endocrine and metabolic diseases, endemic joint diseases, etc.^11^, ^12^ Because primary OA will develop into secondary OA in most cases, it is difficult to distinguish between primary and secondary OA in the clinical treatment. Despite many research advances, the etiology and pathogenesis of OA are still unclear. The main causes of OA include age, obesity, genetics, endocrine and metabolic illnesses, inflammation, trauma, and the combined influence of various factors.^13^, ^14^, ^15^ A deeper comprehension of the OA's pathogenic mechanisms will encourage the identification of new treatment targets to slow the disease progression (Figure 2).

#### 3.1.1 Age

Numerous studies have indicated that age is one of the most prominent causes of OA onset and development. Chronic persistent inflammation, mitochondrial dysfunction, senescent chondrocytes, and alterations in the extracellular matrix are some of the reasons for aging‐related dysfunction.^17^, ^18^ Moreover, aging‐caused higher levels of interleukin‐6 (IL‐6) aggravate the development of OA. Epidemiological studies show that the IL‐6 level is positively correlated with the development of OA.^19^, ^20^, ^21^ Experiment evidence from the mouse model indicates that the IL‐6 gene knockout mice have a more severe spontaneous OA compared with the control.^22^ Mitochondrial dysfunction is associated with almost all aging‐related diseases, including OA.^23^, ^24^ Besides, the dysfunction of mitochondria triggers oxidative stress, chondrocyte apoptosis, and cartilage matrix calcification, leading to cartilage degradation and the cause of OA. Hence, improving mitochondrial dysfunction to reduce cartilage degradation is a new therapeutic target for drug discovery. Chondrocytes derived from the elderly show the characteristics of cellular senescence, such as shortened telomere, increased matrix metalloenzyme activity, cell apoptosis, and decreased growth factor production, related to OA stages. Besides, increasing age also gave rise to alterations in the cartilage extracellular matrix, such as the reduction of collagen (COL), glycosaminoglycan (GAG), and chondroitin sulfate (CS). These changes will weaken the cartilage resiliency and hydration, cause matrix calcification, and contribute to the development of OA.^25^ Understanding the aging‐related factors in OA can help us better understand the molecular mechanisms of OA and then identify new medication targets.

#### 3.1.2 Obesity

Obesity is another risk factor for OA.^26^ Epidemiological investigation shows that obese individuals have a higher incidence of OA than normal‐weight individuals.^27^, ^28^ The pathogenesis of obesity‐related OA is the consequence of a combination of multiple factors.^28^, ^29^ Firstly, obese individuals with excess fat can secrete more inflammatory cytokines into the systemic circulation. The higher levels of inflammation, such as tumor necrosis factor α (TNF‐α), IL‐6, or C‐reactive protein (CRP), promote the progression of OA.^20^ Also, the adipose tissue of obese individuals secretes adipokines, such as leptin and adiponectin. The secreted leptin and adiponectin can modulate immune function, stimulate pro‐inflammatory cytokines production (e.g., TNF‐α and IL‐6), increase the matrix metalloproteinases (MMPs) activity, and promote the generation of nitric oxide. Meanwhile, the mechanical stimulation of the joint surface increases the expression of COX‐2, IL‐1, and MMPs, promoting cartilage degradation.^30^, ^31^ Moreover, obesity can induce the generation of reactive oxygen species (ROS) or advanced glycation end products (AGEs), which can cause death and degeneration of articular chondrocytes and enhance the pathological process of OA.^32^, ^33^

#### 3.1.3 Genetic factor

Although multiple factors are involved in the pathogenesis of OA, genetic factors play a key role in the development of OA.^34^, ^35^ Osteoarthritis is not a monogenic disease but is associated with interactions of multiple genes. The genetic factor associated with OA may be associated with structural defects in joints, alterations in cartilage or bone metabolism, and inflammatory responses. Previous studies have indicated that the related synthesis of matrix protein genes, such as type II procollagen (COL2AI), cartilage matrix protein (CRTM) and cartilage link protein (CRTL l), and insulin‐like growth factor 1 (IGF‐1), is involved in the pathogenesis of OA.^36^ At the same time, the vitamin D receptor gene (VDR) and estrogen receptor alpha gene (ESR1) affect the progression of OA by encoding bone density‐related proteins.^37^, ^38^ Besides, various inflammatory cytokines (e.g., IL‐1, COX‐2, IL‐6, and IL‐10^39^, ^40^), growth factors (e.g., bone morphogenetic protein 2 [BMP‐2] and BMP‐5, human growth differentiation factor [GDF‐5]^41^), proteases, and related inhibitors (e.g., deintegrin metalloproteinase 12 [ADAM12]^42^ and tetranectin [TNA]^43^) are also associated with OA. Wingless/Integrated (WNT),^44^ transforming growth factor β (TGF‐β),^45^ and Notch^44^ signaling pathways are also involved in OA progression.

#### 3.1.4 Trauma

Joint trauma affects all joint tissues, and articular cartilage damage is most pronounced during OA progression.^13^ The most common reasons leading to joint trauma include chondral and osteochondral damage, articular fracture, ligamentous injuries, and meniscal lesions. The incidence of OA increases with patients' age and joint trauma injury time. Since the damage to the articular cartilage caused by joint trauma is irreversible, the articular cartilage is a significant sign of the late stages of OA. Moreover, mechanical trauma triggers enhanced cellular metabolism, accumulation of ROS, and activation of matrix degradation enzymes and inflammatory mediators of joints. Besides, mechanical injury also induces cell death in cartilage and suppresses the synthesis of COL and GAG. The ideal therapy for joint trauma should be multifaceted, including stimulating intrinsic cartilage repair and inhibiting chondrocyte death and matrix loss.

### 3.2 Traditional clinical treatment

**FIGURE 3:**
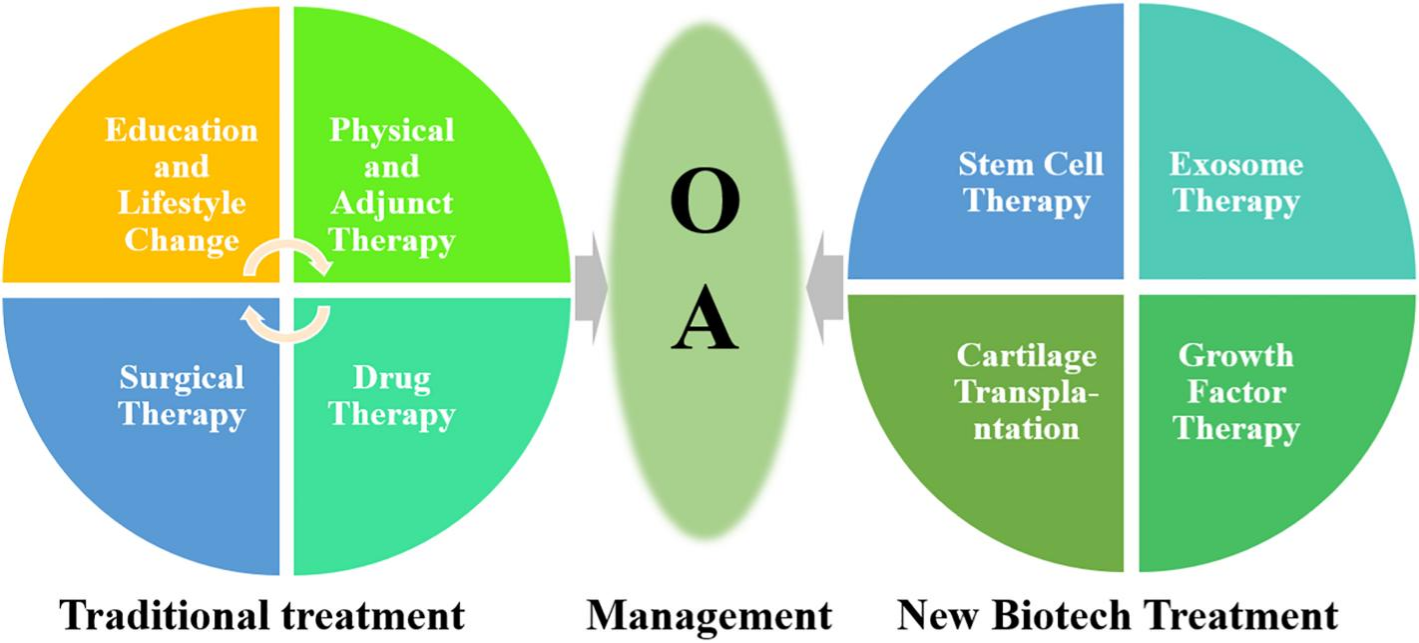
Clinical Osteoarthritis management. Traditional treatments include education, lifestyle changes, physical and adjunct therapy, drug therapy, and surgical medication. New biotech treatments include stem cell therapy, exosome therapy, cartilage transplantation, growth factor therapy, etc.

Most treatments of OA aim to relieve pain and improve joint function. Specific treatment strategies were adopted for different stages of OA progression. Nondrug treatments, such as lifestyle changes, physical therapy, and exercises, are suitable for early‐stage OA patients, which aim to relieve pain symptoms and mental burden. The main goals of drug therapy are anti‐inflammatory drugs, analgesic lubrication, and slowing disease progression. Anti‐inflammatory drugs, local analgesics, and joint lubricants can relieve symptoms to a certain extent by oral or local injection but cannot effectively promote regeneration or articular cartilage change. Surgical treatment is suitable for patients with advanced OA, purposing to relieve pains, correct deformity, prevent further aggravation of joint damage, and improve joint functions. In this section, we will review the traditional treatments of OA, including education and lifestyle changes, physical and adjunct therapy, drug therapy, and surgical medication (Figure 3).

#### 3.2.1 Education and lifestyle changes

OA imposes not only a heavy financial burden but also tremendous mental pressure on patients and families. Education and psychological counseling will relieve their tension and help them to change lifestyles and cooperate with other further therapies.^46^ In the early stages, changing lifestyles to lose weight effectively relieves symptoms and reduces the risk of developing symptomatic OA, especially for obese patients. Additionally, activities combined with appropriate exercise, focused on improving muscle strength and aerobic capacity, can significantly improve symptoms and benefit cardiovascular health.

#### 3.2.2 Physical and adjunct therapy

Thermal modalities, laser therapy, ultrasound therapy, and electrical stimulation acupuncture are the common physical interventions for OA.^46^, ^47^, ^48^ Although physical interventions can be used to support other OA treatments, the effects of these strategies vary from person to person. Numerous factors can affect the responsiveness of individuals with OA, such as treatment modality, compliance, and intensity. Women favor receiving heat treatments, which can significantly enhance their physical and subjective quality of life. Men prefer cold treatments to reduce discomfort and enhance physical performance. However, the evidence to support the effectiveness of modalities (i.e., cold or heat treatment) in OA treatments is unclear.^48^ Laser therapy, ultrasound therapy, and electrical stimulation can relieve OA symptoms by stimulating tissue metabolism and regulating inflammation. Besides, traditional acupuncture is the most common complementary and alternative therapy to relieve pain in various painful diseases, including OA.^49^, ^50^ The underlying mechanism of acupuncture in alleviating pain involves anti‐inflammatory drugs and antioxidants.

#### 3.2.3 Drug therapy

Drug treatment is an effective nonsurgical way to alleviate OA patients' symptoms.^51^, ^52^ In clinical treatment, paracetamol or nonsteroidal anti‐inflammatory drugs are the most commonly used drugs for articular inflammation inhibition and cartilage damage retardation. Frequent oral administration may cause severe gastrointestinal reactions, systemic drug exposure, and cardiovascular side effects. However, intra‐articular injection can maintain a high drug concentration at the target tissues and reduce systemic side effects. In the previous work, hyaluronic acid (HA) and phospholipids were injected as a joint lubricant and drug delivery vehicle to suppress inflammation and maintain synovial joint mobility.^53^, ^54^, ^55^ In addition, MMP inhibitors^56^, ^57^ and antioxidants^57^, ^58^ have also been used to prevent extracellular matrix degradation, stimulate cartilage repair, improve motor functions, and delay the progression of OA.

#### 3.2.4 Surgical therapy

Surgical treatment will be considered when conservative treatments fail, especially for end‐stage OA patients.^59^ Surgical treatments include arthroscopic lavage and debridement, osteotomy, and joint replacement.^60^, ^61^ Arthroscopic lavage and debridement can relieve symptoms of OA by removing synovitis‐causing debris and inflammatory cytokines. In addition, debridement can rebuild the joint surface, trim the meniscus, clean up adhesions, and relieve contractures. However, arthroscopy's benefits in treating knee OA are controversial. Osteotomy is an effective method used in the clinical treatment of OA with varus or valgus deformity, which can reduce pain and improve joint functions. Joint replacement is an effective treatment option for end‐stage OA patients. However, compared with the conservative treatments, clinicians should be aware of more extended hospital stays, higher morbidity, and mortality for surgical patients, especially the elderly.

### 3.3 New biotech treatment

Although traditional OA therapies can alleviate symptoms, relieve pain, and improve motor functions, no absolute safe and effective OA treatment strategies currently exist in clinical treatment.^62^, ^63^ With the development of biotechnology, new OA treatment strategies, such as cell therapy, exosome therapy, cartilage transplantation, growth factor therapy, and platelet‐rich plasma, have been developed for OA treatment.^64^ This section will introduce the new biotech treatments applied in OA treatment (Figure 3).

#### 3.3.1 Stem cell therapy

Stem cells with unlimited self‐renewal ability can differentiate into different cell types, including cartilage cells.^65^ Different from the traditional treatments, stem cell therapy appears to provide a permanent solution for OA treatment. Among the various stem cells, mesenchymal stem cell (MSC) is an ideal choice for OA stem cell therapy due to its wide range of cell sources and excellent cell differentiation potential. In previous works, MSC therapy was successfully used in different animal OA models such as caprine,^66^ rabbits,^67^ dogs,^68^ and mice.^69^ The mechanisms of improved OA treatment are attributed to meniscal tissue regeneration, tissue metabolism restoration, and inflammation inhibition. In addition to the results of animal OA models, many clinical trials also show that MSC treatment can relieve pains, promote meniscal tissue regeneration, and improve motor functions. However, stem cell therapies in clinical OA treatment still face many challenges, such as limited MSC sources, unstable cell proliferation, potential tumorigenicity, and high cost.

#### 3.3.2 Exosome therapy

Exosomes are vesicles ranging between 40 and 150 nm with multiple cellular activities, such as cells' microenvironment remodeling and signal transduction.^70^ Exosomes derived from stem cells showed similar therapeutic effects compared with parental stem cells when used for the treatment of a variety of diseases including OA. In recent years, numerous studies have shown that exosomes derived from MSCs have exact therapeutic effects on OA and cartilage damage.^71^, ^72^, ^73^ This is because the secretion of MSCs can enhance cell viability and promote endogenous cell regeneration. The main effects of its OA treatment mechanism include promoting cartilage formation, accelerating tissue repair, regulating inflammatory response, and maintaining chondrocyte homeostasis.^74^, ^75^ Compared with stem cell therapy, exosomes derived from stem cells have similar effects to parental stem cells.^76^ Exosomes do not have cellular structures, which can avoid immune rejection and have good biological safety and efficacy. Moreover, stem cell‐derived exosomes are safe for autologous or allogeneic therapy, avoiding pathogen infectivity, genomic variation, and teratogenicity of stem cell therapy. Besides, compared with stem cell therapy, exosome therapies have high safety, easy preservation, less side effects, and few ethical issues.^74^, ^77^ However, the production, enrichment, efficient separation, and targeted delivery of exosomes still face many problems.

#### 3.3.3 Cartilage transplantation

Cartilage transplantation is an effective treatment of OA. There are three common cartilage transplantation methods: autologous osteochondral transplantation, allogeneic osteochondral transplantation, and cartilage transplantation based on tissue engineering techniques.^78^, ^79^ Autologous cartilage transplantation is suitable for patients with minor cartilage defects, and allogeneic transplantation is often used for large cartilage defects. However, cartilage graft therapy may fail when the defect is too large. It is worth mentioning that traditional articular chondrocyte transplantation is combined with biomaterials and bioactive molecules to provide an effective tissue‐engineered articular surface for successful long‐term function.

#### 3.3.4 Growth factor therapy

Recent studies have shown that some production factors, such as fibroblast growth factor (FGF), platelet‐derived growth factor (PDGF),^80^ epidermal growth factor (EGF),^81^ and TGF‐β,^82^ have specific biological activities for the proliferation and regeneration of cartilage and anti‐apoptosis. Besides, the platelet‐rich plasma can effectively treat arthritis by angiogenesis, regulation of articular cartilage anabolism, and stem cell recruitment.^83^, ^84^, ^85^, ^86^

## 4 BIOMATERIALS FOR OA TREATMENT

**FIGURE 4:**
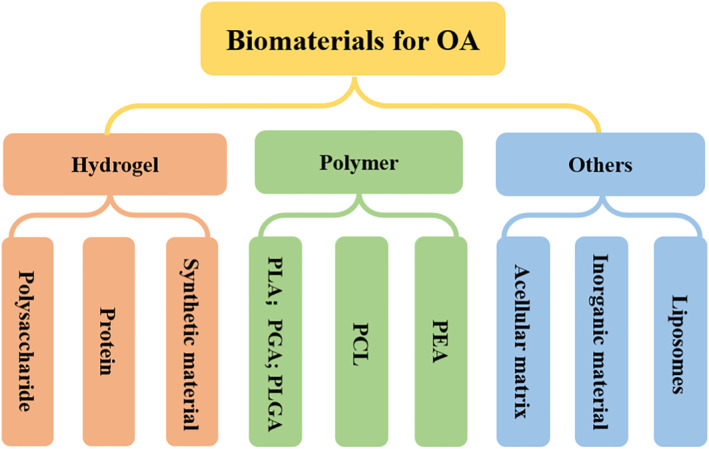
Biomaterials applied for osteoarthritis treatment include polysaccharide hydrogel, natural protein hydrogel, synthetic hydrogel, synthetic polymer, acellular matrix, inorganic materials, liposomes, etc.

Articular cartilage has limited self‐repair ability after injury. Adjuvant therapy based on biomaterials can effectively improve the self‐repair ability of articular cartilage, showing great therapeutic potential in recent years. This section will introduce various biomaterials commonly used in OA therapy, such as hydrogels, non‐hydrogel polymers, and inorganic nanomaterials (Figure 4).

### 4.1 Hydrogel

Hydrogel is a water‐rich polymer formed by physical or chemical cross‐linking of hydrophilic polymers, which has similar characteristics to the extracellular matrix, and is widely used in drug delivery, cell encapsulation, and tissue engineering.^87^, ^88^, ^89^, ^90^ Hydrogels can be divided into natural materials and synthetic materials depending on their origin.^88^, ^91^ Most naturally derived polymers have weak mechanical properties and rapid degradation with significant batch differences. In contrast, synthetic materials' biological, physical, and mechanical properties can be chemically modified. Natural material‐based hydrogels with better biocompatibility were classified into polysaccharides and proteins. Polysaccharides mainly include chitosan (Chi), alginates (Alg), hyaluronic acid (HA), heparin (Hep), CS, dextran (Dex),^88^, ^92^, ^93^, ^94^ etc. Proteins mainly contain collagen (Col), gelatin (Gel), fibrin (Fib), and silk protein (Sil),^95^, ^96^, ^97^ etc. Meanwhile, synthetic hydrogels currently used in OA treatment include poly (ethyleneglycol) (PEG) and poly (N‐isopropylacrylamide) (PNIPAM).

#### 4.1.1 Polysaccharide

**FIGURE 5:**
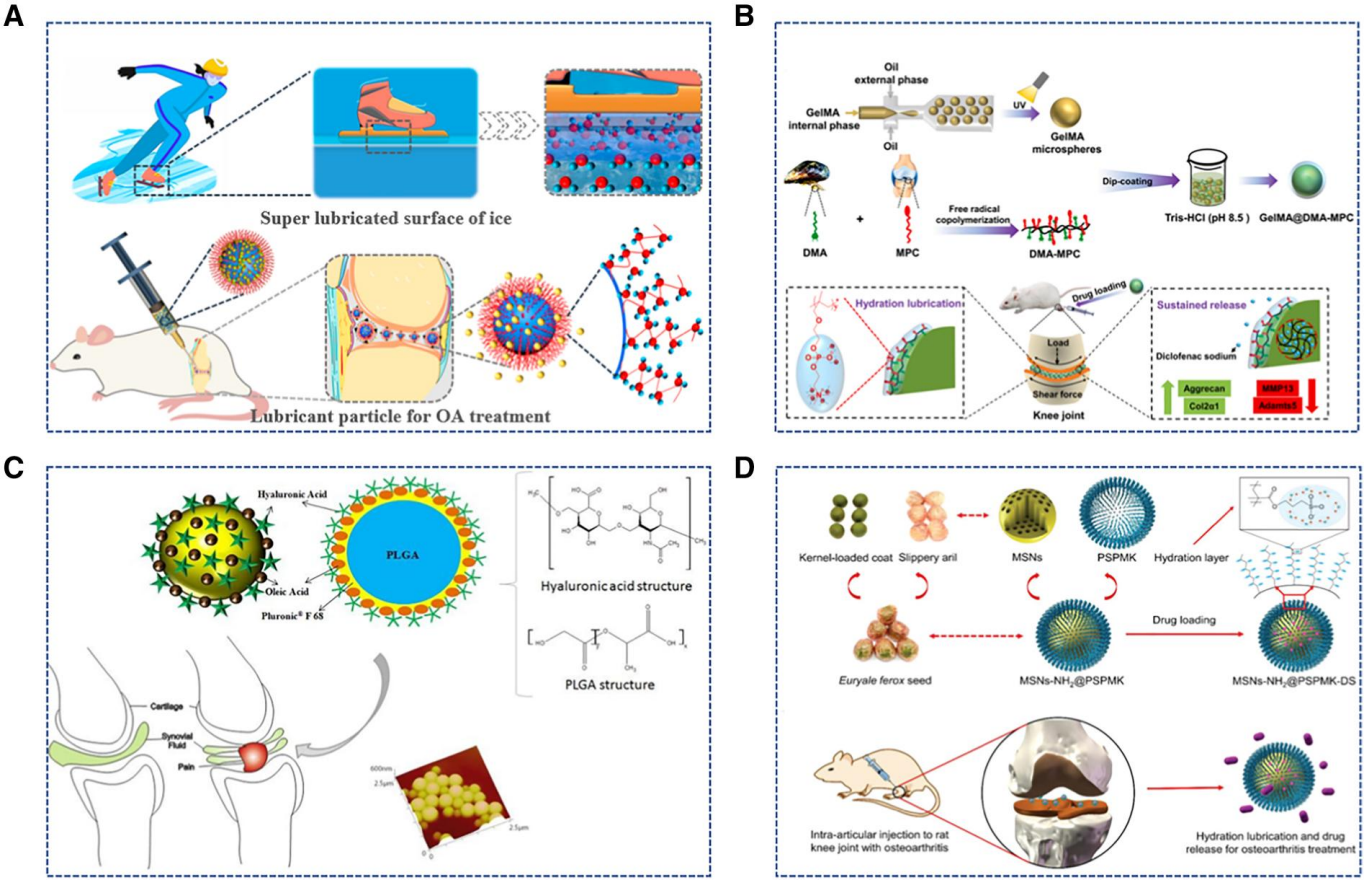
Biomaterials for Osteoarthritis treatment. (A) Ice‐inspired lubricant drug delivery particle derived from HA. Reproduced with permission.^63^ Copyright 2021, American Chemical Society. (B) GelMA microspheres with enhanced lubrication and controllable drug release. Reproduced with permission.^52^ Copyright 2021, The Authors, published by Elsevier. (C) HA‐modified LGA particles. Reproduced with permission.^118^ Copyright 2019, Elsevier. (D) Euryale ferox seed‐inspired superlubricated nanoparticles. Reproduced with permission.^131^ Copyright 2019, John Wiley and Sons.

Some natural polysaccharides (e.g., CS and HA) are the intrinsic components of the joint, which have a natural biological activity, such as lubrication, anti‐inflammatory, and antioxidant.^98^, ^99^, ^100^ CS, either alone or in combination with other materials, has a demonstrated benefit for OA treatment. Clinical data suggest that oral or injected CS can reduce pain and inhibit cartilage degeneration by reducing OA inflammation.^98^, ^101^, ^102^ Moreover, CS‐based drug sustained release systems have been used for OA treatment. For example, previous work used a solvent injection method to prepare the aceclofenac‐loaded CS for the treatment of OA. The prepared particles exhibited satisfactory drug release performance in vitro and excellent drug uptake ability at the knee joint in vivo, making it an effective drug delivery for OA treatment.^103^ Because of excellent lubrication, HA is the major component of synovial membranes^100^ and is often used as a lubricant to reduce pains in patients.^104^ Moreover, HA can also improve joint function by inhibiting inflammatory factor expression and promoting matrix protein synthesis.^105^ However, HA degrades rapidly *in vivo*, which limits its application as a joint lubricant and drug delivery vehicle.^62^ Previous work has developed photo‐cross‐linkable methacrylate anhydride HA (HAMA) particles for drug delivery and OA treatment (Figure 5A). In this study, HA was modified by methacrylate anhydride, which improved mechanical properties, delayed degradation rate, and enhanced the drug release ability of HA. These particles based on HAMA can be furtherly modified by phosphorylcholine to enhance lubricity and inhibit inflammatory responses after diclofenac sodium loading.^63^ Chi has a similar structure to chondroitin glycosaminoglycan with cationic properties and high charge density.^106^ A previous work applied HA and Chi to construct nanoparticles for nucleic acid delivery vectors in the treatment of OA. These HA/Chi nanoparticles can effectively load plasmid vectors and improve the transfection efficiency of plasmids in primary chondrocytes.^107^

#### 4.1.2 Protein

Like natural polysaccharides hydrogels, natural protein hydrogels, such as Gel, Sil, and their derivatives, have been used to treat OA. Han et al. prepared injectable hydrogel microspheres with enhanced lubrication and controlled drug release properties by microfluidic technology.^52^ These hydrogel microspheres are based on photo‐cross‐linked methacrylate Gel (GelMA) with anti‐inflammatory drugs. In vitro experiments showed that GelMA hydrogel microspheres have excellent lubricity and drug release properties (Figure 5B). The results of animal experiments confirmed that intra‐articular injection of functionalized microspheres into the knee joint of OA model rats could effectively inhibit articular cartilage lesions and improve the therapeutic effect.^52^ Sil is also widely used in various tissue engineering applications due to its good mechanical stability and bioactivity. Previously, Gel/Sil hybrid microspheres were prepared by the water‐in‐oil emulsion technique to load curcumin for OA treatment.^108^ The diameters of the microspheres prepared by the water‐in‐oil method range from 100 to 300 µm. The Gel/Sil hybrid microspheres greatly slow down the drug's rate of degradation compared to only Gel microspheres. In addition, curcumin‐loaded Gel/Sil hybrid microspheres significantly reduced inflammation and effectively inhibited the progression of OA in animal studies.^108^ Fib is a common histocompatibility natural protein produced during coagulation, which can effectively promote tissue regeneration.^109^ Fib hydrogels can be rapidly generated by directly mixing Fib and thrombin, applied for human adipose stem cells (hASCs) loading. High concentrations of Fib can induce hASCs to maintain a round morphology in vitro, similar to natural chondrocytes. In animal transplantation experiments, this Fib gels can effectively promote the differentiation of hASCs into chondrocytes.^110^

#### 4.1.3 Synthetic materials

Compared to natural hydrogel materials, synthetic materials have better mechanical properties and batch stability. In recent years, synthetic hydrogels, such as PEG and PNIPAM, have also been used in OA. Yang et al. prepared HA/PEG hydrogels by Diels‐Alder loading with MSC‐derived exosomes for OA treatment.^111^ This HA/PEG hydrogel is cytocompatibility, which can effectively achieve the sustained release of MSC‐derived exosomes and keep the therapeutic functions of exosomes. Animal studies have shown that this exosome‐loaded HA/PEG hydrogel can slow the release of exosomes, which is more effective than the exosome treatment group alone.^111^ In a previous study, Zhang et al. synthesized drug‐delivery nanospheres with enhanced lubrication and temperature response based on PNIPAM and poly (2‐methacryloyloxyethyl phosphorylcholine) (PMPC). This PNIPAM‐PMPC microsphere has excellent lubrication properties, drug retardation properties, and cellular biocompatibility. The animal experiments confirmed that this kind of PNIPAM‐PMPC nanospheres could effectively inhibit chondrocyte degeneration and thus alleviate the osteoarthritic process.^112^ In addition, Yang et al. developed a kind of sustained‐release microsphere based on the anti‐opal structure of PNIPAM, which has good temperature response properties and can achieve smart drug release when OA occurs.^62^

### 4.2 Polymers

Polymers are compounds with macromolecules with relative molecular masses of several thousand or even several million. With the development of science and technology, a wide variety of macromolecular monomers have emerged, and polymeric materials are widely used in the construction of artificial organs, tissue engineering, drug delivery, and medical devices. In this section, we will briefly introduce the application of synthetic polymers in OA treatments.

#### 4.2.1 Polylactic acid and polyglycolic acid

Polylactic acid (PLA) is a polyester obtained by polymerizing lactic acid as building blocks.^113^ PLA can be used alone or with other materials to produce sustained‐release carriers for OA treatments. For example, Liu et al. synthesized adenosine‐loaded PLA‐b‐PEG nanospheres by emulsification. These nanospheres continuously release adenosine to maintain intra‐articular homeostasis and prevent the development of OA.^114^ Polyglycolic acid (PGA), a simple polyester with excellent biodegradability and biocompatibility, has various medical applications, including cartilage tissue engineering. For example, Mahmoudifar et al. induced hASCs to chondrocytes on a PGA scaffold. Cells on PGA scaffolds showed higher COL and GAG levels than the control group, indicating that the interactions of cell and scaffold can influence the differentiation of stem cells and promote extracellular matrix production.^115^ Poly lactic‐co‐glycolic acid (PLGA) is a biocompatible and biodegradable copolymer of PLA and PGA, which has been used in drug sustained release and tissue engineering. Ko et al. developed a kind of joint cavity‐injectable PLGA microspheres. Sulforaphane‐loaded microspheres could effectively inhibit the progression of surgically induced OA in rats.^116^ Moreover, PLGA microspheres can also be used for gene therapy for OA. For example, Li et al. developed PLGA nanoparticles loaded with siRNA targeting p47phox, effectively inhibiting ROS production and alleviating chondrocyte death.^117^ In addition, Mota et al. developed nanoparticles composed of PLGA and HA with good drug‐retarding properties, lubricity, and hemocompatibility. Animal experiments demonstrated that HA/PLGA nanoparticles could effectively inhibit inflammation^118^ (Figure 5C).

#### 4.2.2 Poly (caprolactone)

Polycaprolactone (PCL), also known as poly ε‐caprolactone, is a synthetic polymer with biocompatibility and biodegradability, widely used in drug carriers and tissue engineering.^119^ Arunkumar et al. prepared viscoelastic PCL‐CS hybrid gels by dispersing PCL microparticles in Chi solution.^120^ In the experiments on cells and animals, PCL‐CS hybrid gel showed good biocompatibility, drug release ability, and OA therapeutic effects.^120^ Liang et al. developed a nanofiber made of a copolymer of PCL and PCL‐grafted lignin.^121^ Lignin provides intrinsic antioxidant activity in this nanofiber system, while PCL modulates the mechanical properties. In vitro cellular experiments demonstrated that this composite nanofiber could inhibit ROS effectively and alleviate chondrocyte apoptosis induced by oxidative stress. This composite nanofiber can effectively reduce OARSI scores of rabbit OA caused by papain after 4 weeks of treatment.^121^ In another study, Su et al. synthesized a pH‐responsive amphiphilic PEG‐hydrazone‐PCL nano‐micelles copolymer, which can be used to load Kartogenin (KGN) for the treatment of OA.^122^

#### 4.2.3 Poly (ester amide)

Polyester amide (PEA) is a new type of biodegradable material with high thermomechanical properties and mechanical strength.^123^ In previous works, PEA was also used for developing drug delivery vehicles for OA treatment. Rudnik‐Jansen et al. prepared PEA microspheres with an average diameter of 22.4 μm by ultrasonic emulsification. These PEA microspheres can sustainedly release triamcinolone acetonide (TAA) for more than 60 days in vitro. Animal experiments indicated that these drug‐loaded microspheres could be retained in the joint cavity for 70 days and effectively inhibit inflammation of OA.^124^ Villamagna et al. developed a PEA nanoparticle delivering PPARδ antagonist GSK3787, which is approximately 600 nm in diameter and can be loaded with 8% GSK3787 for continuous release in 30 days. In addition, this nanoparticle has good biocompatibility and can be continuously retained in the joint cavity for 7 days.^125^ Tellegen et al. prepared PEA microspheres loaded with COX‐2 inhibitor celecoxib. The microspheres exhibited sustained drug release retardation ability for 28 days. After injected into rats, the celecoxib‐loaded microspheres have no systemic or local adverse effects and can effectively reduce OA.^126^

### 4.3 Other materials

In addition to the most common materials mentioned above, there are other biomaterials used in the treatment of OA, such as acellular matrix materials (dECM), inorganic materials, and liposomes.

#### 4.3.1 Acellular matrix materials

Extracellular matrix (ECM), a complex produced by cellular secretion, has a variety of bioactive substances, promoting cells' proliferation, migration, and adhesion.^127^ Removal of cellular components can effectively reduce the immunogenicity of ECM, making it applicable in various tissue engineering applications, including OA treatment. Cartilage‐derived dECM was used to treat a rabbit model of OA, promoting COL and GAG production in arthritic rabbits' cartilage tissue without serious complications, such as infection and skin necrosis.^128^ In addition, the combination of human umbilical cord blood‐derived MSCs (hUCB‐MSCs) and cartilage dECM effectively improved the motor status and histological assessment of articular cartilage in OA goats.^129^

#### 4.3.2 Inorganic materials

Inorganic nanoparticle (INP) is a new kind of nanomaterial with good biocompatibility and a large specific surface area, widely used to prepare various drug delivery carriers.^91^, ^130^ Recently, a novel poly (3‐sulfopropyl methacrylate potassium salt)‐coated mesoporous silica nanoparticles (MSNs‐NH2@PSPMK) was synthesized by Yan et al (Figure 5D). The nanoparticles effectively reduce the friction between joint surfaces by the grafted PSPMK polyelectrolyte polymer. Because of the mesoporous channels, nanoparticles could load and release anti‐inflammatory drugs. The in vitro and in vivo experiments have demonstrated that this nanoparticle drug delivery vehicle could effectively inhibit chondrocyte degeneration.^131^ Moreover, magnetic nanoparticles can also assist in treating articular cartilage diseases. For example, Jafari et al. developed PEG‐coated magnetic nanoparticles to improve drug delivery efficiency. At the alternating magnetic fields, the magnetic nanoparticles can transport deep into articular cartilage to promote cartilage repair.^132^

#### 4.3.3 Liposomes

Liposomes, spherical vesicles with phospholipid bilayers, have been recently investigated for drug delivery and gene therapy.^133^ Ji et al. constructed a liposomal drug delivery system based on 1,2‐distearoyl‐sn‐glycero‐3‐phosphocholine (DSPC) loaded with D‐glucosamine sulfate (GAS) for lubrication and anti‐inflammatory.^134^ This biocompatible liposome drug delivery system can promote chondrocytes' proliferation. The in vitro cellular assays demonstrated that this DSPC liposome inhibited the expression of inflammatory factors and effectively avoided tumor necrosis factor α (TNF‐α)‐induced chondrocyte degeneration.^134^ In addition, liposomes can also be combined with other biomaterials for the treatment of OA. For example, Zhang et al. constructed HA‐Lipo‐DIC/DEX nanoparticles by mixing the dexamethasone and diclofenac‐loaded liposomes with HA. Inflammation in the joints of OA mice was decreased by the drug's continuous release from the nanoparticles for more than 168 h.^135^

## 5 CONCLUSIONS AND OUTLOOK

Osteoarthritis is a disabling disease that seriously affects human health. Numerous biologists, clinicians, and material scientists have long worked on this disease in their respective fields. However, most of them are confined to their respective fields and lack knowledge and communication in other fields, which undoubtedly limits the resolution of OA problems. Therefore, this review briefly explains the biological pathogenesis and current clinical treatments of OA. In more detail, we present most of the therapeutics in the animal testing stage. We hope this review will contribute to understanding OA in different fields and facilitate the final resolution of OA treatment. In addition, we also present our views and insights on biomaterials applied in the treatment of OA.

Although various biomaterials and their derivatives are widely used in preparing drug delivery vehicles for OA treatment, their clinical applications are limited by several challenges. First, most of the biomaterials used for OA treatment are generated in the laboratory. These biomaterials may be expensive, difficult to mass produce, or face batch instability problems. Second, these biosafety and clinical efficacy tests are mostly based on animal studies, which need further evaluation in clinical trials to obtain approvals from the relevant regulatory authorities. After this, these biomaterials are expected to be widely used in clinical practice.

In the future, with the deepening understanding of OA and continuous improvement of material technology, it is expected that the combination of traditional physical therapy, drug therapy, surgical therapy, novel biotech therapy, and biomaterial technology will provide new solutions for OA.

## AUTHOR CONTRIBUTIONS

Fangfu Ye conceived the conceptualization and designed the paper. Yang Lei, Qingfei Zhang, Gaizheng Kuang, and Xiaochen Wang wrote the paper. Qihui Fan participated in discussion.

## CONFLICT OF INTEREST

The authors declare that they have no known competing financial interests or personal relationships that could have appeared to influence the work reported in this paper. Fangfu Ye is a member of the *Smart Medicine* editorial board.
